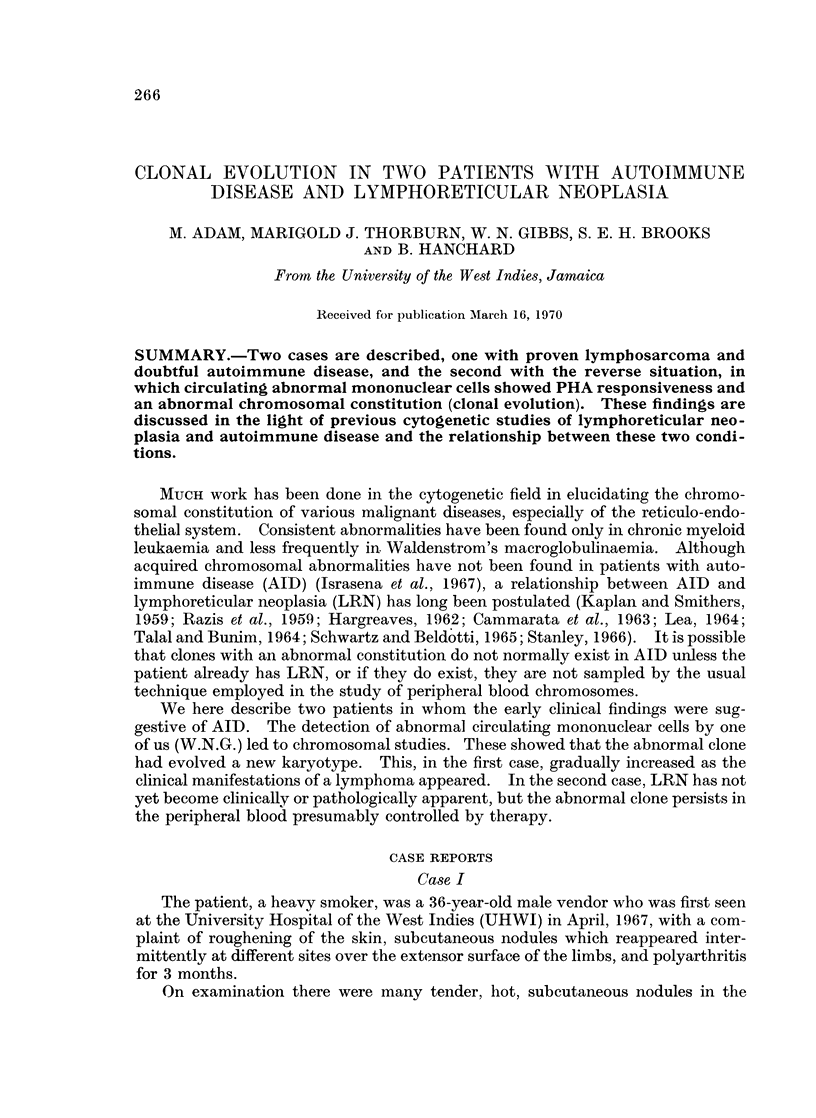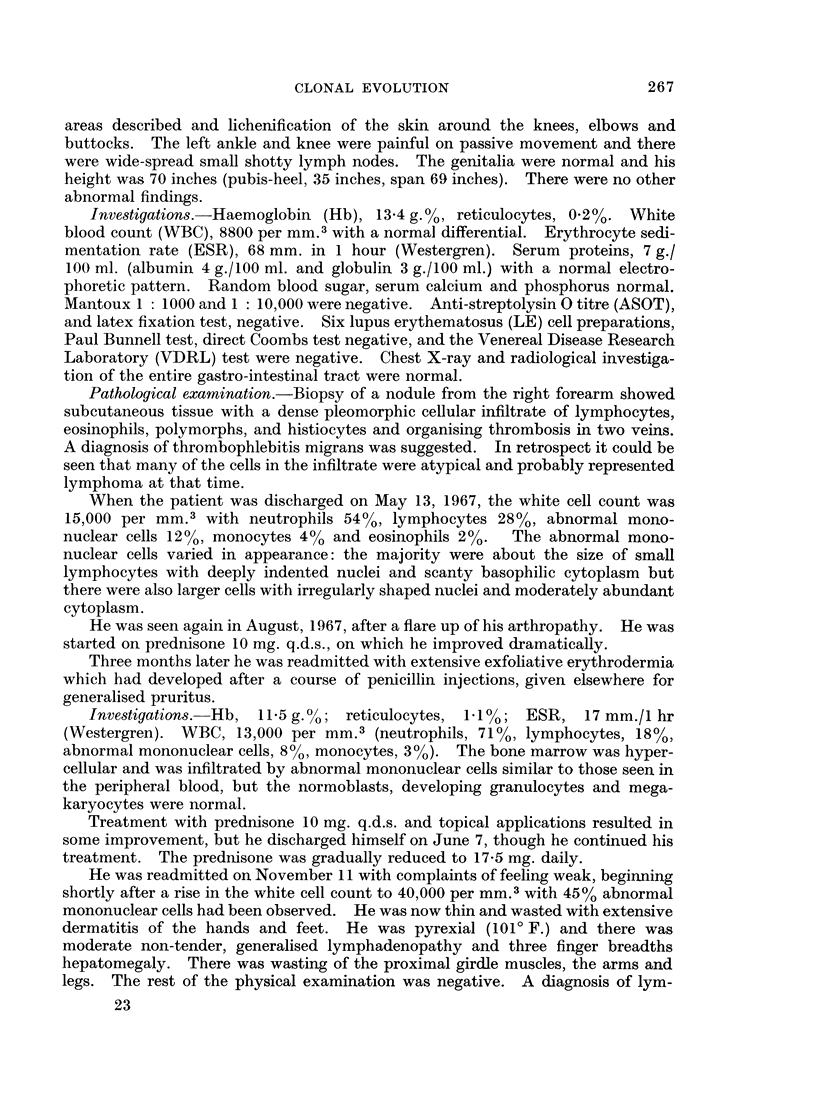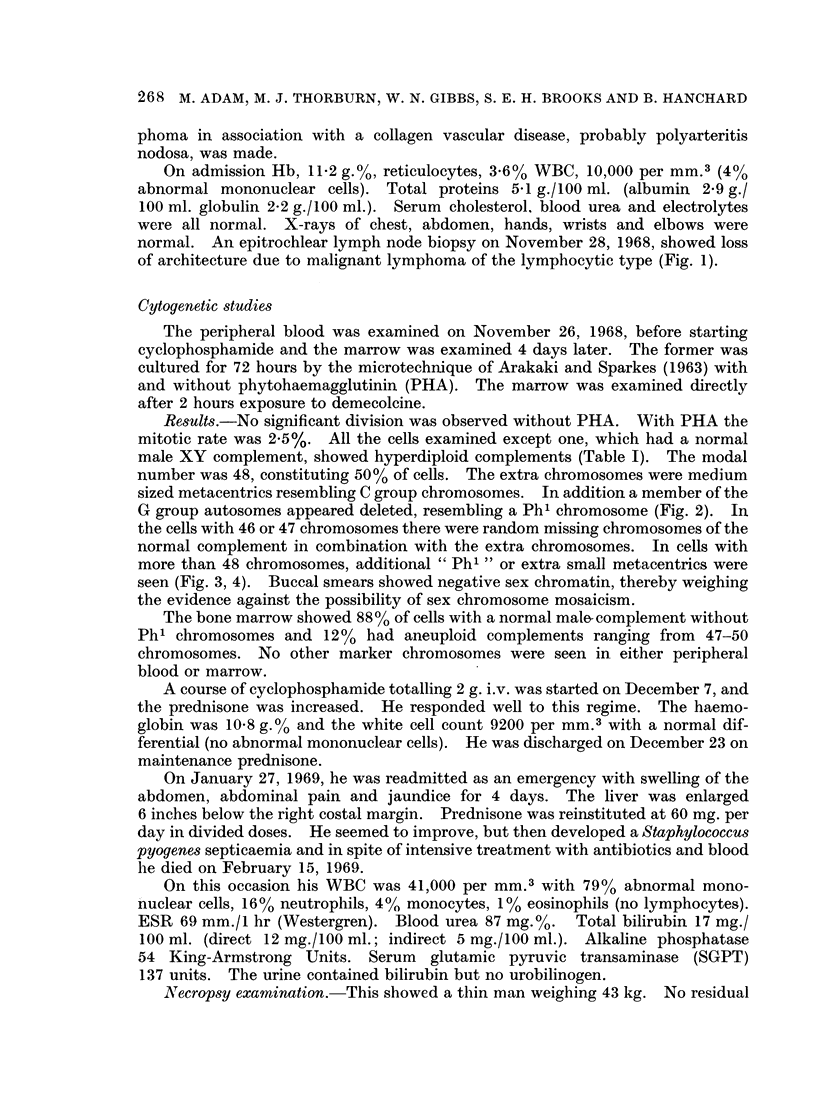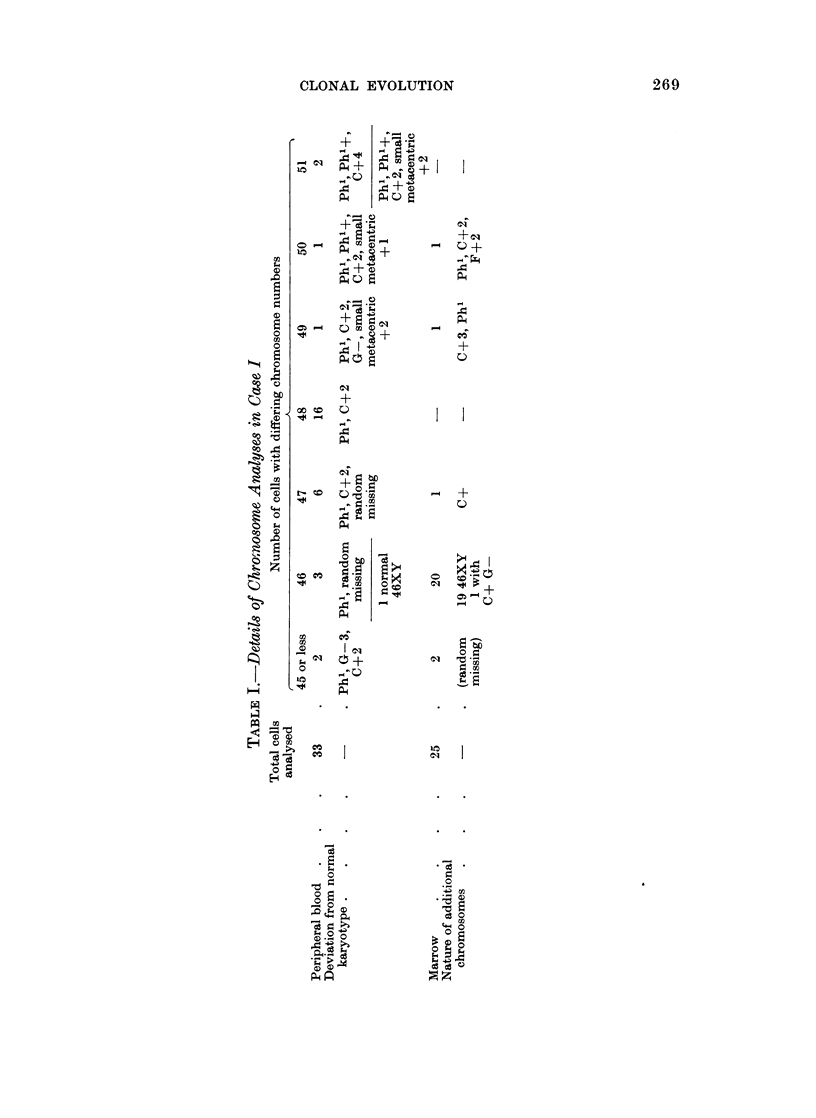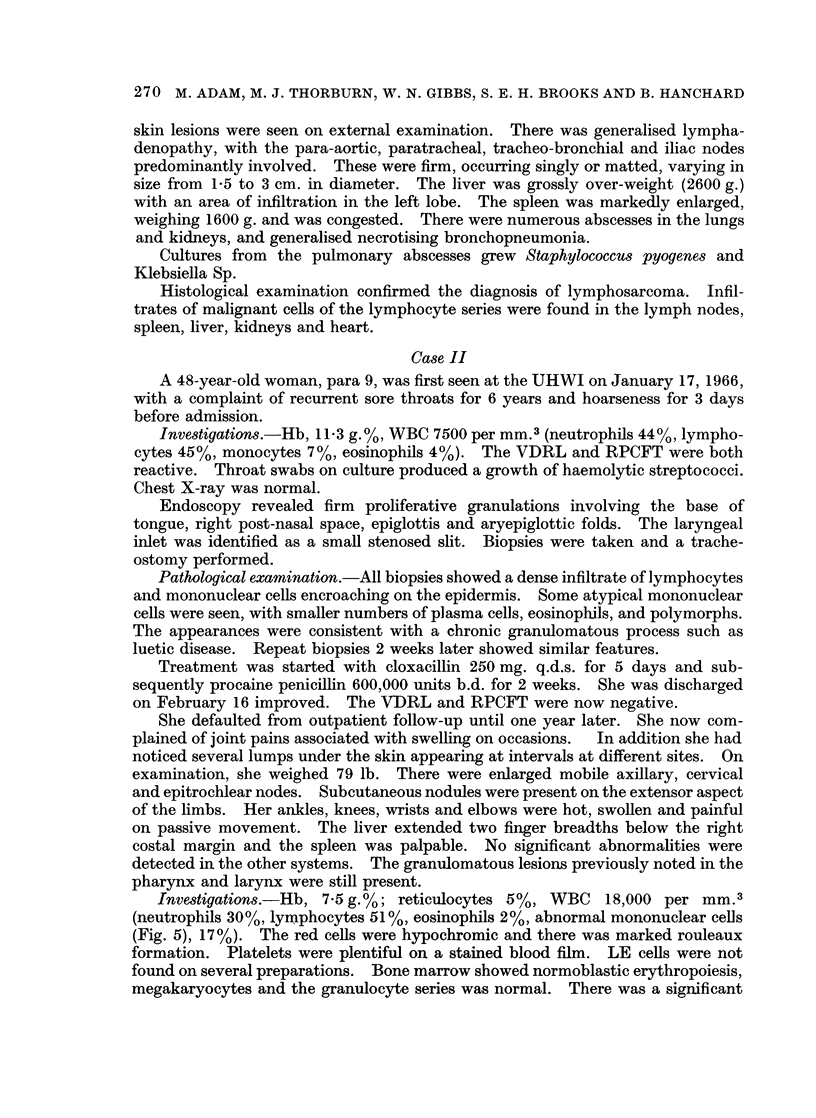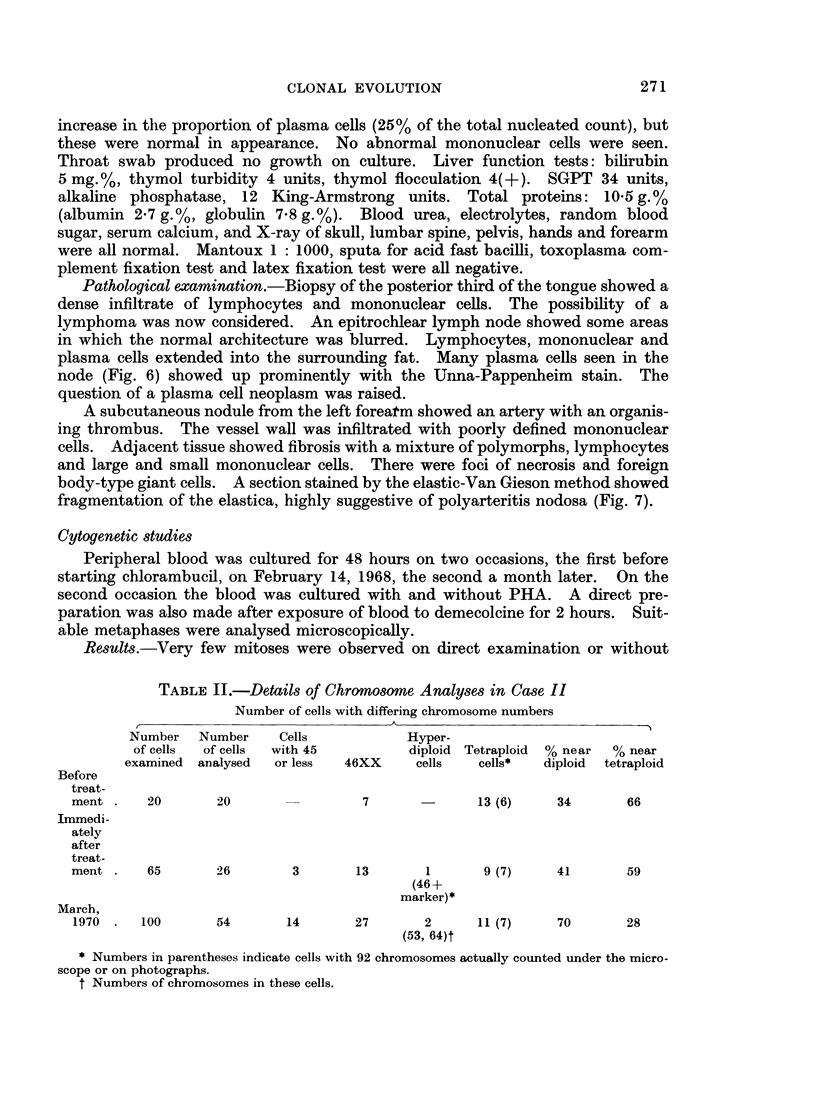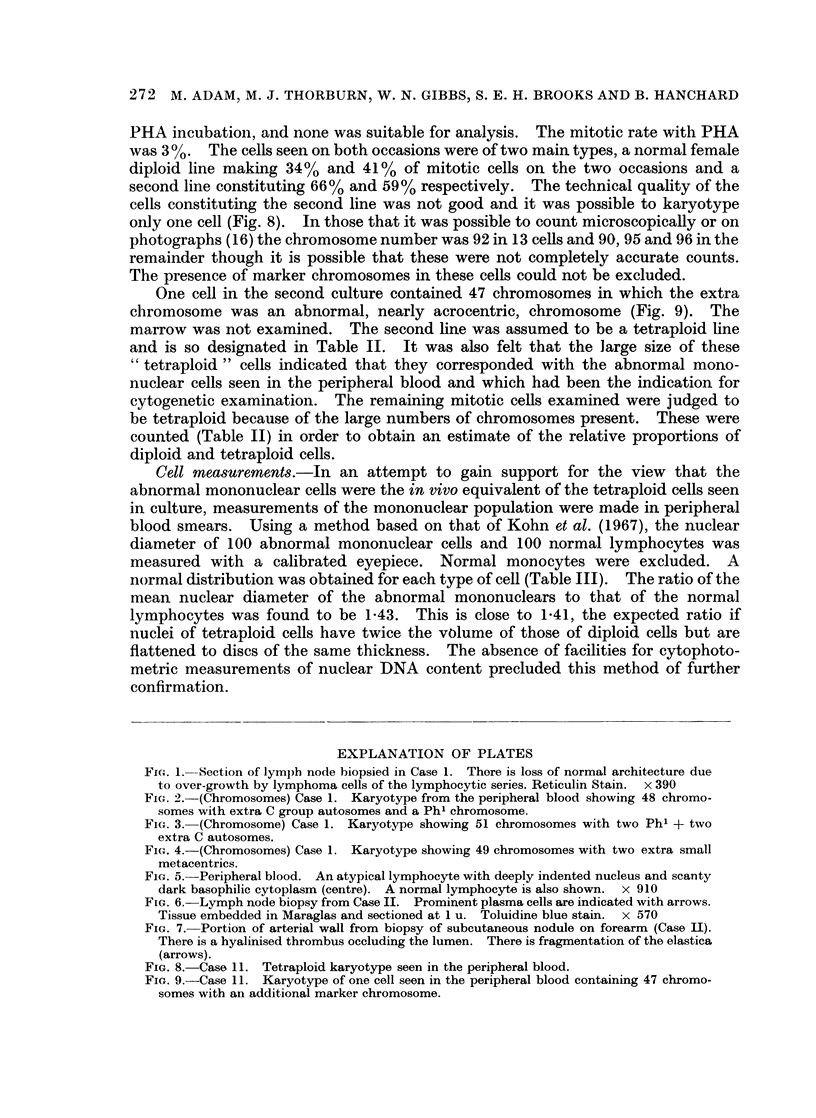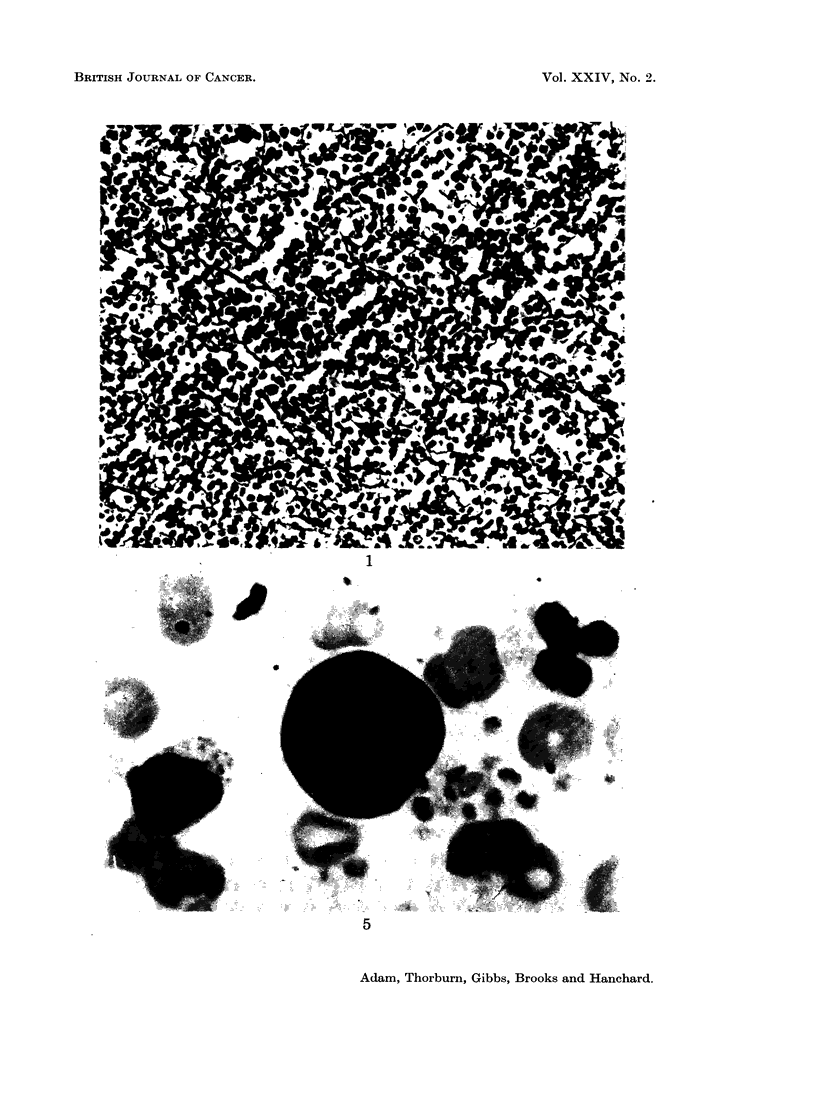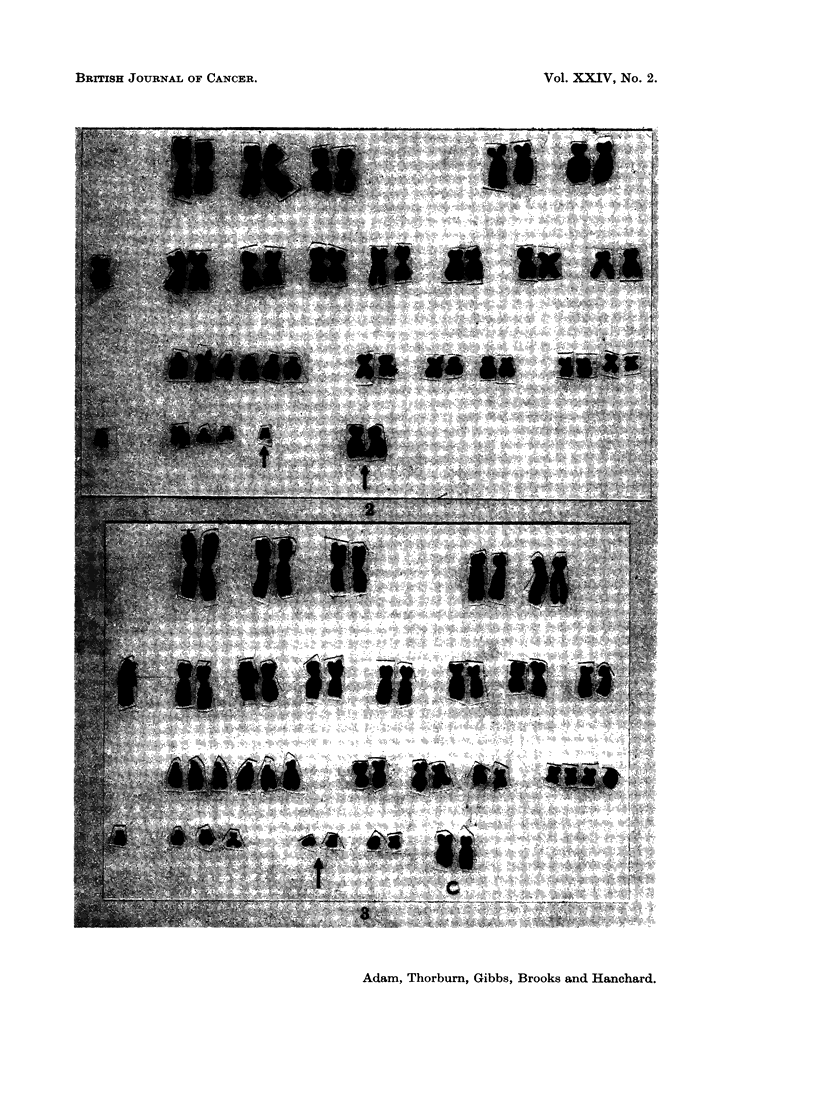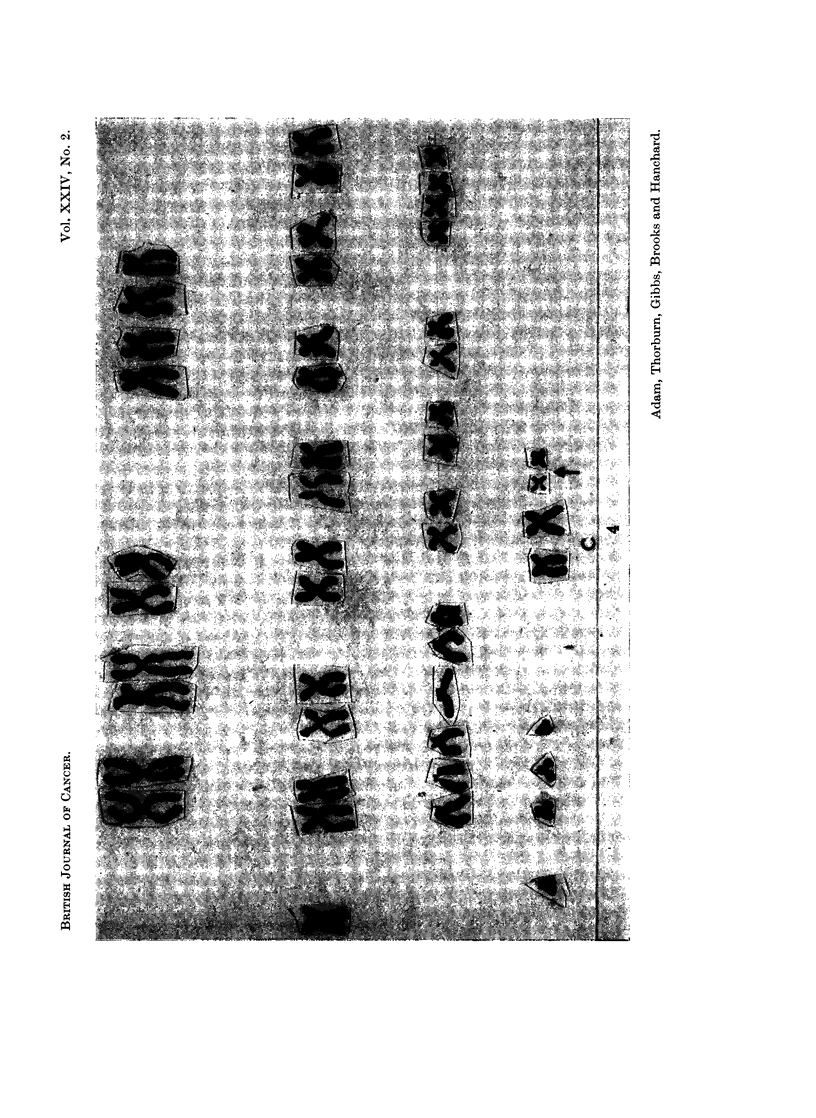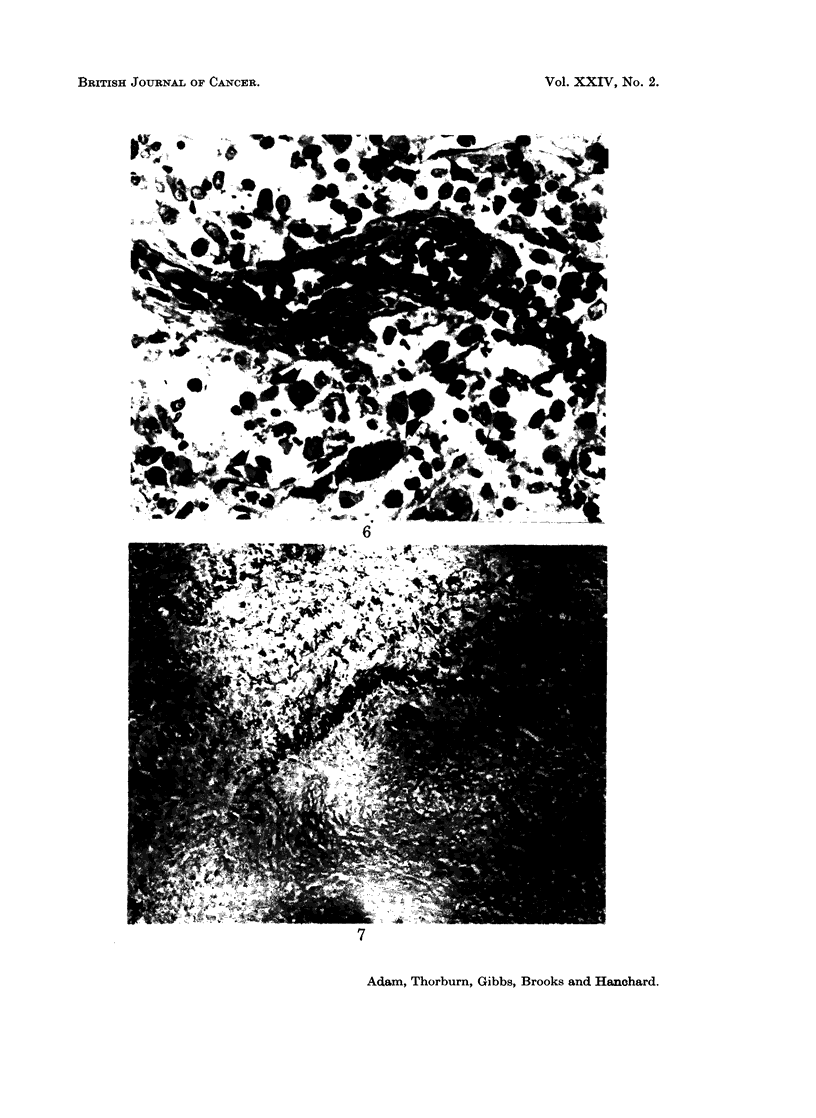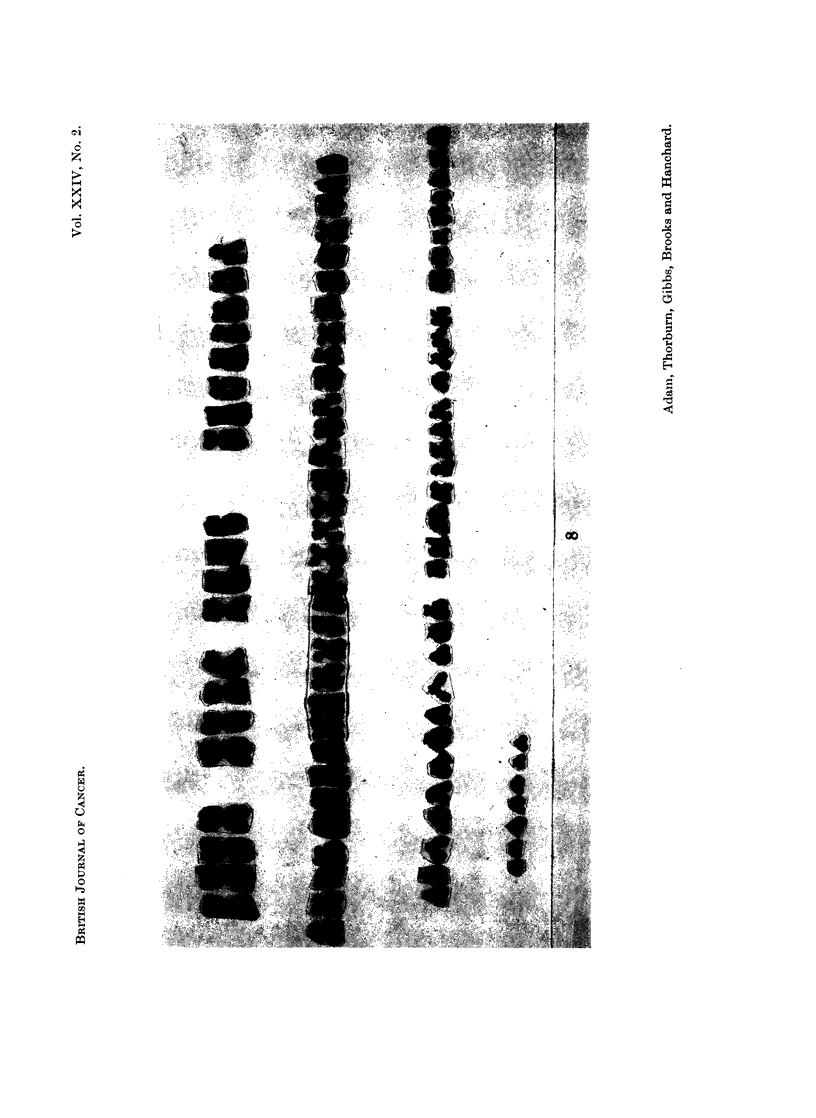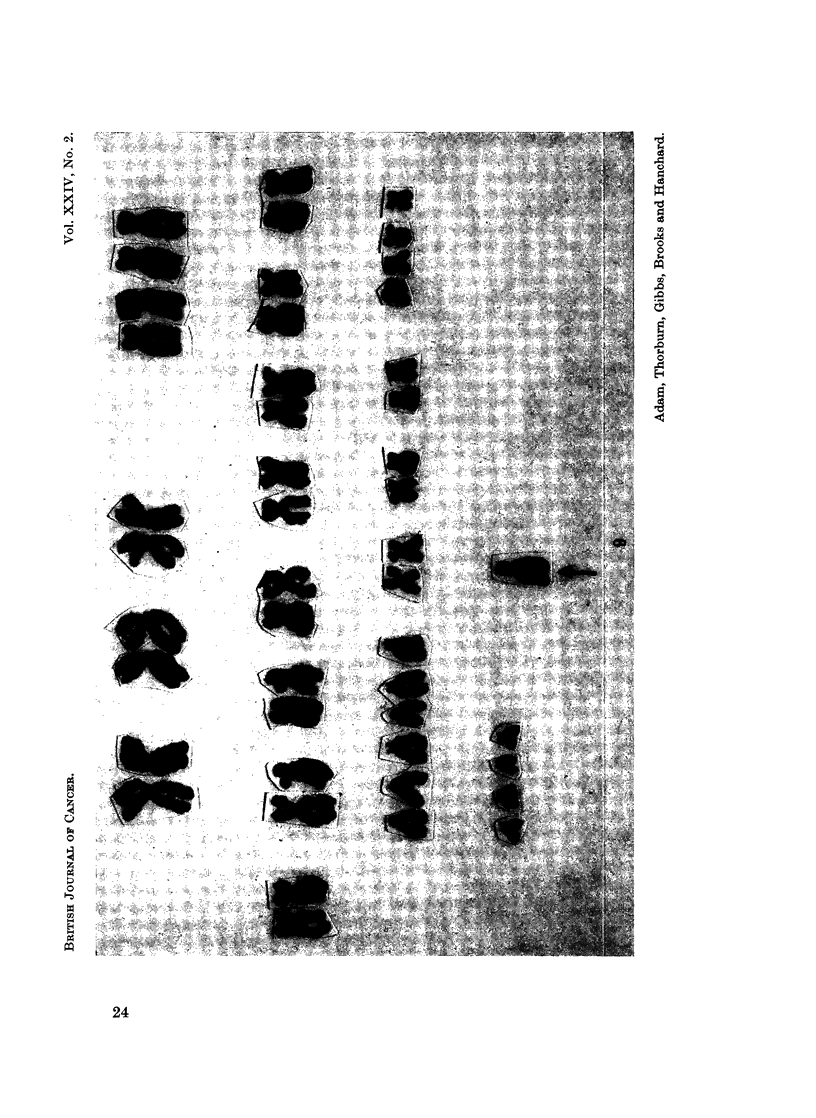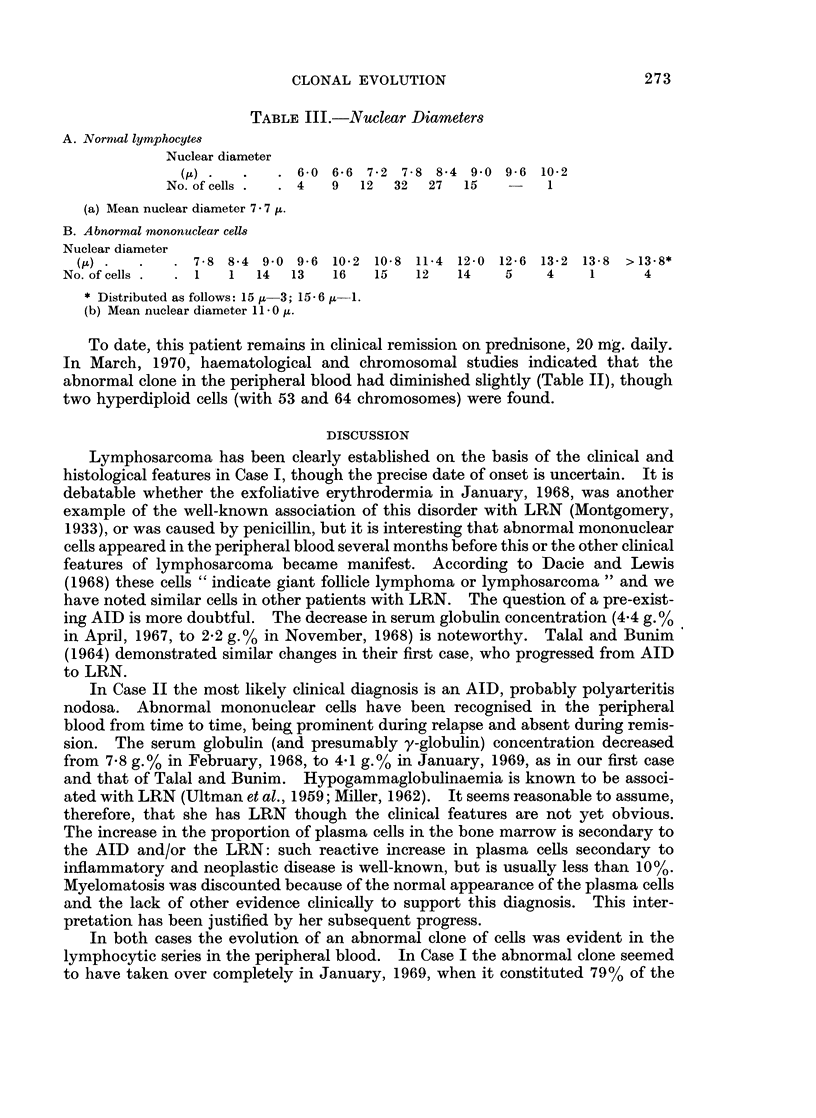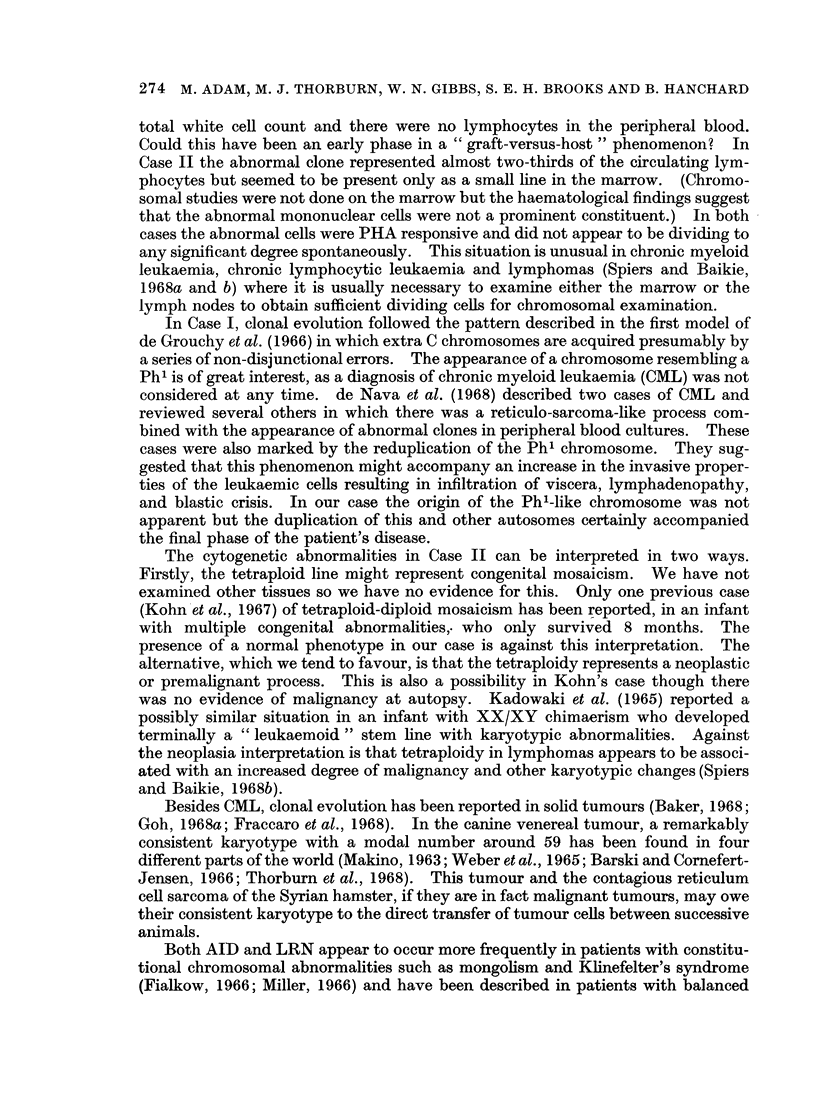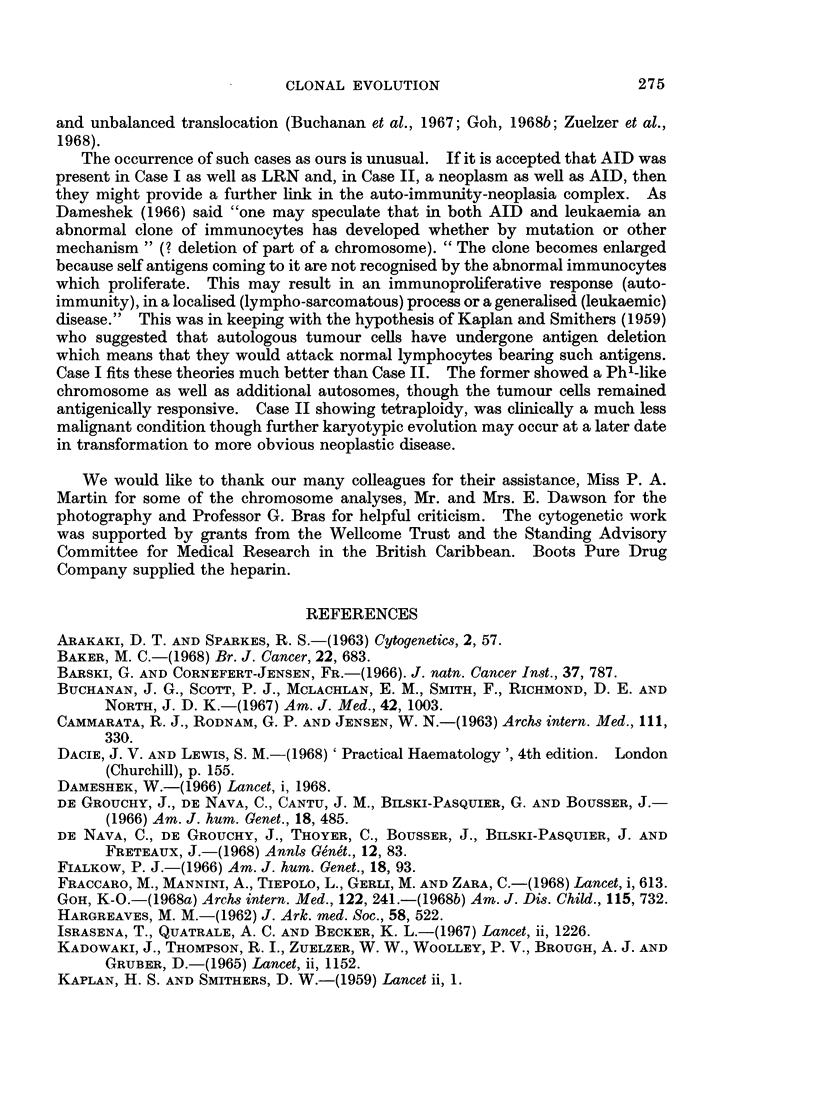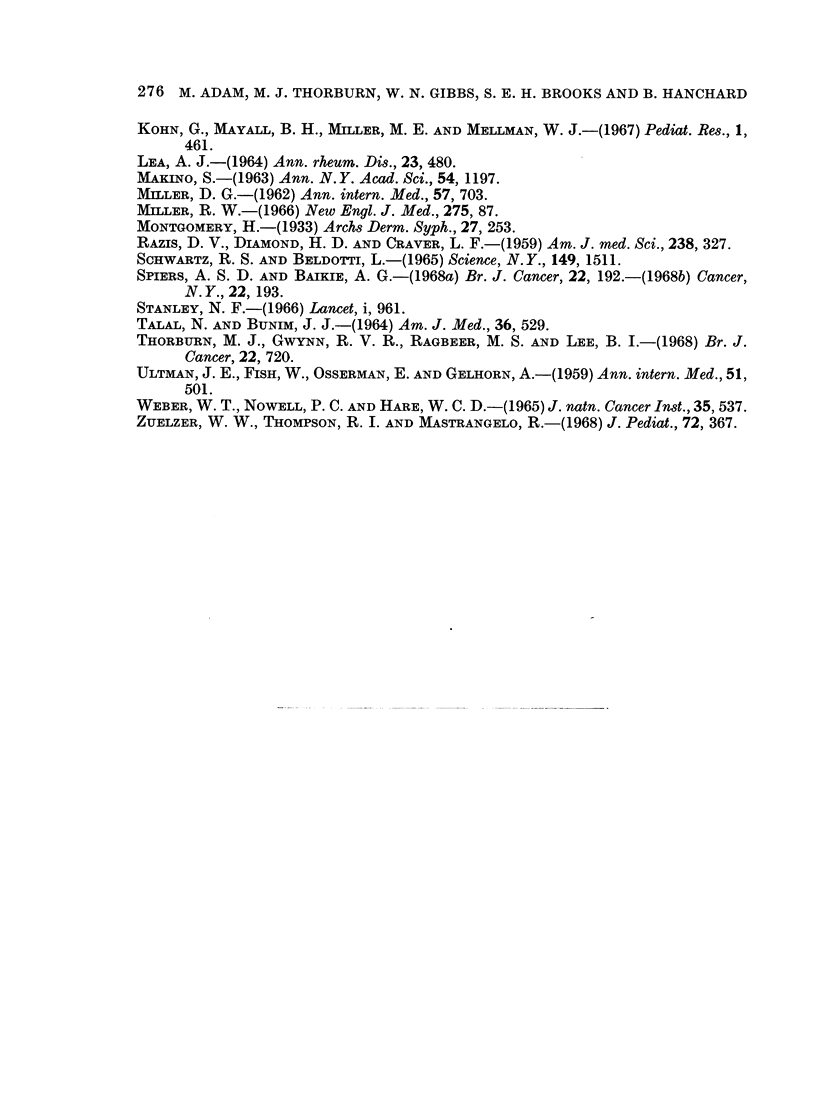# Clonal Evolution in Two Patients with Autoimmune Disease and Lymphoreticular Neoplasia

**DOI:** 10.1038/bjc.1970.30

**Published:** 1970-06

**Authors:** M. Adam, Marigold J. Thorburn, W. N. Gibbs, S. E. H. Brooks, B. Hanchard

## Abstract

**Images:**


### CLONAL EVOLUTION IN TWO PATIENTS WITH AUTOIMMUNE DISEASE AND LYMPHORETICULAR NEOPLASIA

M. ADAM, MARIGOLD J. THORBURN, W. N. GIBBS, S. E. H. BROOKS AND B. HANCHARD

*From the University of the West Indies, Jamaica*

Received for publication March 16, 1970

**SUMMARY.—Two cases are described, one with proven lymphosarcoma and doubtful autoimmune disease, and the second with the reverse situation, in which circulating abnormal mononuclear cells showed PHA responsiveness and an abnormal chromosomal constitution (clonal evolution). These findings are discussed in the light of previous cytogenetic studies of lymphoreticular neoplasia and autoimmune disease and the relationship between these two conditions.**

Much work has been done in the cytogenetic field in elucidating the chromosomal constitution of various malignant diseases, especially of the reticulo-endothelial system. Consistent abnormalities have been found only in chronic myeloid leukaemia and less frequently in Waldenstrom's macroglobulinaemia. Although acquired chromosomal abnormalities have not been found in patients with autoimmune disease (AID) (Israsena *et al.*, 1967), a relationship between AID and lymphoreticular neoplasia (LRN) has long been postulated (Kaplan and Smithers, 1959; Razis *et al.*, 1959; Hargreaves, 1962; Cammarata *et al.*, 1963; Lea, 1964; Talal and Bunim, 1964; Schwartz and Beldòtti, 1965; Stanley, 1966). It is possible that clones with an abnormal constitution do not normally exist in AID unless the patient already has LRN, or if they do exist, they are not sampled by the usual technique employed in the study of peripheral blood chromosomes.

We here describe two patients in whom the early clinical findings were suggestive of AID. The detection of abnormal circulating mononuclear cells by one of us (W.N.G.) led to chromosomal studies. These showed that the abnormal clone had evolved a new karyotype. This, in the first case, gradually increased as the clinical manifestations of a lymphoma appeared. In the second case, LRN has not yet become clinically or pathologically apparent, but the abnormal clone persists in the peripheral blood presumably controlled by therapy.

### CASE REPORTS

### *Case I*

The patient, a heavy smoker, was a 36-year-old male vendor who was first seen at the University Hospital of the West Indies (UHWI) in April, 1967, with a complaint of roughening of the skin, subcutaneous nodules which reappeared intermittently at different sites over the extensor surface of the limbs, and polyarthritis for 3 months.

On examination there were many tender, hot, subcutaneous nodules in the areas described and lichenification of the skin around the knees, elbows and buttocks. The left ankle and knee were painful on passive movement and there were wide-spread small shotty lymph nodes. The genitalia were normal and his height was 70 inches (pubis-heel, 35 inches, span 69 inches). There were no other abnormal findings.

*Investigations.*—Haemoglobin (Hb), 13·4 g.%, reticulocytes, 0·2%. White blood count (WBC), 8800 per mm.$^3$ with a normal differential. Erythrocyte sedimentation rate (ESR), 68 mm. in 1 hour (Westergren). Serum proteins, 7 g./100 ml. (albumin 4 g./100 ml. and globulin 3 g./100 ml.) with a normal electrophoretic pattern. Random blood sugar, serum calcium and phosphorus normal. Mantoux 1 : 1000 and 1 : 10,000 were negative. Anti-streptolysin O titre (ASOT), and latex fixation test, negative. Six lupus erythematosus (LE) cell preparations, Paul Bunnell test, direct Coombs test negative, and the Venereal Disease Research Laboratory (VDRL) test were negative. Chest X-ray and radiological investigation of the entire gastro-intestinal tract were normal.

*Pathological examination.*—Biopsy of a nodule from the right forearm showed subcutaneous tissue with a dense pleomorphic cellular infiltrate of lymphocytes, eosinophils, polymorphs, and histiocytes and organising thrombosis in two veins. A diagnosis of thrombophlebitis migrans was suggested. In retrospect it could be seen that many of the cells in the infiltrate were atypical and probably represented lymphoma at that time.

When the patient was discharged on May 13, 1967, the white cell count was 15,000 per mm.$^3$ with neutrophils 54%, lymphocytes 28%, abnormal mononuclear cells 12%, monocytes 4% and eosinophils 2%. The abnormal mononuclear cells varied in appearance: the majority were about the size of small lymphocytes with deeply indented nuclei and scanty basophilic cytoplasm but there were also larger cells with irregularly shaped nuclei and moderately abundant cytoplasm.

He was seen again in August, 1967, after a flare up of his arthropathy. He was started on prednisone 10 mg. q.d.s., on which he improved dramatically.

Three months later he was readmitted with extensive exfoliative erythrodermia which had developed after a course of penicillin injections, given elsewhere for generalised pruritus.

*Investigations.*—Hb, 11·5 g.%; reticulocytes, 1·1%; ESR, 17 mm./1 hr (Westergren). WBC, 13,000 per mm.$^3$ (neutrophils, 71%, lymphocytes, 18%, abnormal mononuclear cells, 8%, monocytes, 3%). The bone marrow was hypercellular and was infiltrated by abnormal mononuclear cells similar to those seen in the peripheral blood, but the normoblasts, developing granulocytes and megakaryocytes were normal.

Treatment with prednisone 10 mg. q.d.s. and topical applications resulted in some improvement, but he discharged himself on June 7, though he continued his treatment. The prednisone was gradually reduced to 17·5 mg. daily.

He was readmitted on November 11 with complaints of feeling weak, beginning shortly after a rise in the white cell count to 40,000 per mm.$^3$ with 45% abnormal mononuclear cells had been observed. He was now thin and wasted with extensive dermatitis of the hands and feet. He was pyrexial (101° F.) and there was moderate non-tender, generalised lymphadenopathy and three finger breadths hepatomegaly. There was wasting of the proximal girdle muscles, the arms and legs. The rest of the physical examination was negative. A diagnosis of lymphoma in association with a collagen vascular disease, probably polyarteritis nodosa, was made.

On admission Hb, 11·2 g.%, reticulocytes, 3·6% WBC, 10,000 per mm.$^3$ (4% abnormal mononuclear cells). Total proteins 5·1 g./100 ml. (albumin 2·9 g./100 ml. globulin 2·2 g./100 ml.). Serum cholesterol, blood urea and electrolytes were all normal. X-rays of chest, abdomen, hands, wrists and elbows were normal. An epitrochlear lymph node biopsy on November 28, 1968, showed loss of architecture due to malignant lymphoma of the lymphocytic type (Fig. 1).

*Cytogenetic studies*

The peripheral blood was examined on November 26, 1968, before starting cyclophosphamide and the marrow was examined 4 days later. The former was cultured for 72 hours by the microtechnique of Arakaki and Sparkes (1963) with and without phytohaemagglutinin (PHA). The marrow was examined directly after 2 hours exposure to demecolcine.

*Results.*—No significant division was observed without PHA. With PHA the mitotic rate was 2·5%. All the cells examined except one, which had a normal male XY complement, showed hyperdiploid complements (Table I). The modal number was 48, constituting 50% of cells. The extra chromosomes were medium sized metacentrics resembling C group chromosomes. In addition a member of the G group autosomes appeared deleted, resembling a $Ph^1$ chromosome (Fig. 2). In the cells with 46 or 47 chromosomes there were random missing chromosomes of the normal complement in combination with the extra chromosomes. In cells with more than 48 chromosomes, additional " $Ph^1$ " or extra small metacentrics were seen (Fig. 3, 4). Buccal smears showed negative sex chromatin, thereby weighing the evidence against the possibility of sex chromosome mosaicism.

The bone marrow showed 88% of cells with a normal male complement without $Ph^1$ chromosomes and 12% had aneuploid complements ranging from 47–50 chromosomes. No other marker chromosomes were seen in either peripheral blood or marrow.

A course of cyclophosphamide totalling 2 g. i.v. was started on December 7, and the prednisone was increased. He responded well to this regime. The haemoglobin was 10·8 g.% and the white cell count 9200 per mm.$^3$ with a normal differential (no abnormal mononuclear cells). He was discharged on December 23 on maintenance prednisone.

On January 27, 1969, he was readmitted as an emergency with swelling of the abdomen, abdominal pain and jaundice for 4 days. The liver was enlarged 6 inches below the right costal margin. Prednisone was reinstituted at 60 mg. per day in divided doses. He seemed to improve, but then developed a *Staphylococcus pyogenes* septicaemia and in spite of intensive treatment with antibiotics and blood he died on February 15, 1969.

On this occasion his WBC was 41,000 per mm.$^3$ with 79% abnormal mononuclear cells, 16% neutrophils, 4% monocytes, 1% eosinophils (no lymphocytes). ESR 69 mm./1 hr (Westergren). Blood urea 87 mg.%. Total bilirubin 17 mg./100 ml. (direct 12 mg./100 ml.; indirect 5 mg./100 ml.). Alkaline phosphatase 54 King-Armstrong Units. Serum glutamic pyruvic transaminase (SGPT) 137 units. The urine contained bilirubin but no urobilinogen.

*Necropsy examination.*—This showed a thin man weighing 43 kg. No residual

TABLE I.—*Details of Chromosome Analyses in Case I*

| | Total cells analysed | Number of cells with differing chromosome numbers | | | | | | |
|---|---|---|---|---|---|---|---|---|
| | | 45 or less | 46 | 47 | 48 | 49 | 50 | 51 |
| Peripheral blood | 33 | 2 | 3 | 6 | 16 | 1 | 1 | 2 |
| Deviation from normal karyotype | — | $Ph^1$, G−3, C+2 | $Ph^1$, random missing | $Ph^1$, C+2, random missing | $Ph^1$, C+2 | $Ph^1$, C+2, G−, small metacentric +2 | $Ph^1$, $Ph^1$+, C+2, small metacentric +1 | $Ph^1$, $Ph^1$+, C+4 |
| | | | 1 normal 46XY | | | | | $Ph^1$, $Ph^1$+, C+2, small metacentric +2 |
| Marrow | 25 | 2 | 20 | 1 | — | 1 | 1 | — |
| Nature of additional chromosomes | — | (random missing) | 19 46XY 1 with C+ G− | C+ | — | C+3, $Ph^1$ | $Ph^1$, C+2, F+2 | — |

skin lesions were seen on external examination. There was generalised lymphadenopathy, with the para-aortic, paratracheal, tracheo-bronchial and iliac nodes predominantly involved. These were firm, occurring singly or matted, varying in size from 1·5 to 3 cm. in diameter. The liver was grossly over-weight (2600 g.) with an area of infiltration in the left lobe. The spleen was markedly enlarged, weighing 1600 g. and was congested. There were numerous abscesses in the lungs and kidneys, and generalised necrotising bronchopneumonia.

Cultures from the pulmonary abscesses grew *Staphylococcus pyogenes* and Klebsiella Sp.

Histological examination confirmed the diagnosis of lymphosarcoma. Infiltrates of malignant cells of the lymphocyte series were found in the lymph nodes, spleen, liver, kidneys and heart.

*Case II*

A 48-year-old woman, para 9, was first seen at the UHWI on January 17, 1966, with a complaint of recurrent sore throats for 6 years and hoarseness for 3 days before admission.

*Investigations.*—Hb, 11·3 g.%, WBC 7500 per mm.$^3$ (neutrophils 44%, lymphocytes 45%, monocytes 7%, eosinophils 4%). The VDRL and RPCFT were both reactive. Throat swabs on culture produced a growth of haemolytic streptococci. Chest X-ray was normal.

Endoscopy revealed firm proliferative granulations involving the base of tongue, right post-nasal space, epiglottis and aryepiglottic folds. The laryngeal inlet was identified as a small stenosed slit. Biopsies were taken and a tracheostomy performed.

*Pathological examination.*—All biopsies showed a dense infiltrate of lymphocytes and mononuclear cells encroaching on the epidermis. Some atypical mononuclear cells were seen, with smaller numbers of plasma cells, eosinophils, and polymorphs. The appearances were consistent with a chronic granulomatous process such as luetic disease. Repeat biopsies 2 weeks later showed similar features.

Treatment was started with cloxacillin 250 mg. q.d.s. for 5 days and subsequently procaine penicillin 600,000 units b.d. for 2 weeks. She was discharged on February 16 improved. The VDRL and RPCFT were now negative.

She defaulted from outpatient follow-up until one year later. She now complained of joint pains associated with swelling on occasions. In addition she had noticed several lumps under the skin appearing at intervals at different sites. On examination, she weighed 79 lb. There were enlarged mobile axillary, cervical and epitrochlear nodes. Subcutaneous nodules were present on the extensor aspect of the limbs. Her ankles, knees, wrists and elbows were hot, swollen and painful on passive movement. The liver extended two finger breadths below the right costal margin and the spleen was palpable. No significant abnormalities were detected in the other systems. The granulomatous lesions previously noted in the pharynx and larynx were still present.

*Investigations.*—Hb, 7·5 g.%; reticulocytes 5%, WBC 18,000 per mm.$^3$ (neutrophils 30%, lymphocytes 51%, eosinophils 2%, abnormal mononuclear cells (Fig. 5), 17%). The red cells were hypochromic and there was marked rouleaux formation. Platelets were plentiful on a stained blood film. LE cells were not found on several preparations. Bone marrow showed normoblastic erythropoiesis, megakaryocytes and the granulocyte series was normal. There was a significant increase in the proportion of plasma cells (25% of the total nucleated count), but these were normal in appearance. No abnormal mononuclear cells were seen. Throat swab produced no growth on culture. Liver function tests: bilirubin 5 mg.%, thymol turbidity 4 units, thymol flocculation 4(+). SGPT 34 units, alkaline phosphatase, 12 King-Armstrong units. Total proteins: 10·5 g.% (albumin 2·7 g.%, globulin 7·8 g.%). Blood urea, electrolytes, random blood sugar, serum calcium, and X-ray of skull, lumbar spine, pelvis, hands and forearm were all normal. Mantoux 1 : 1000, sputa for acid fast bacilli, toxoplasma complement fixation test and latex fixation test were all negative.

*Pathological examination.*—Biopsy of the posterior third of the tongue showed a dense infiltrate of lymphocytes and mononuclear cells. The possibility of a lymphoma was now considered. An epitrochlear lymph node showed some areas in which the normal architecture was blurred. Lymphocytes, mononuclear and plasma cells extended into the surrounding fat. Many plasma cells seen in the node (Fig. 6) showed up prominently with the Unna-Pappenheim stain. The question of a plasma cell neoplasm was raised.

A subcutaneous nodule from the left forearm showed an artery with an organising thrombus. The vessel wall was infiltrated with poorly defined mononuclear cells. Adjacent tissue showed fibrosis with a mixture of polymorphs, lymphocytes and large and small mononuclear cells. There were foci of necrosis and foreign body-type giant cells. A section stained by the elastic-Van Gieson method showed fragmentation of the elastica, highly suggestive of polyarteritis nodosa (Fig. 7).

### *Cytogenetic studies*

Peripheral blood was cultured for 48 hours on two occasions, the first before starting chlorambucil, on February 14, 1968, the second a month later. On the second occasion the blood was cultured with and without PHA. A direct preparation was also made after exposure of blood to demecolcine for 2 hours. Suitable metaphases were analysed microscopically.

*Results.*—Very few mitoses were observed on direct examination or without

TABLE II.—*Details of Chromosome Analyses in Case II*

| | Number of cells with differing chromosome numbers | | | | | | | |
|---|---|---|---|---|---|---|---|---|
| | Number of cells examined | Number of cells analysed | Cells with 45 or less | 46XX | Hyper-diploid cells | Tetraploid cells* | % near diploid | % near tetraploid |
| Before treatment . | 20 | 20 | — | 7 | — | 13 (6) | 34 | 66 |
| Immediately after treatment . | 65 | 26 | 3 | 13 | 1 (46+ marker)* | 9 (7) | 41 | 59 |
| March, 1970 . | 100 | 54 | 14 | 27 | 2 (53, 64)† | 11 (7) | 70 | 28 |

* Numbers in parentheses indicate cells with 92 chromosomes actually counted under the microscope or on photographs.

† Numbers of chromosomes in these cells.

PHA incubation, and none was suitable for analysis. The mitotic rate with PHA was 3%. The cells seen on both occasions were of two main types, a normal female diploid line making 34% and 41% of mitotic cells on the two occasions and a second line constituting 66% and 59% respectively. The technical quality of the cells constituting the second line was not good and it was possible to karyotype only one cell (Fig. 8). In those that it was possible to count microscopically or on photographs (16) the chromosome number was 92 in 13 cells and 90, 95 and 96 in the remainder though it is possible that these were not completely accurate counts. The presence of marker chromosomes in these cells could not be excluded.

One cell in the second culture contained 47 chromosomes in which the extra chromosome was an abnormal, nearly acrocentric, chromosome (Fig. 9). The marrow was not examined. The second line was assumed to be a tetraploid line and is so designated in Table II. It was also felt that the large size of these "tetraploid" cells indicated that they corresponded with the abnormal mononuclear cells seen in the peripheral blood and which had been the indication for cytogenetic examination. The remaining mitotic cells examined were judged to be tetraploid because of the large numbers of chromosomes present. These were counted (Table II) in order to obtain an estimate of the relative proportions of diploid and tetraploid cells.

*Cell measurements.*—In an attempt to gain support for the view that the abnormal mononuclear cells were the *in vivo* equivalent of the tetraploid cells seen in culture, measurements of the mononuclear population were made in peripheral blood smears. Using a method based on that of Kohn *et al.* (1967), the nuclear diameter of 100 abnormal mononuclear cells and 100 normal lymphocytes was measured with a calibrated eyepiece. Normal monocytes were excluded. A normal distribution was obtained for each type of cell (Table III). The ratio of the mean nuclear diameter of the abnormal mononuclears to that of the normal lymphocytes was found to be 1·43. This is close to 1·41, the expected ratio if nuclei of tetraploid cells have twice the volume of those of diploid cells but are flattened to discs of the same thickness. The absence of facilities for cytophotometric measurements of nuclear DNA content precluded this method of further confirmation.

---

EXPLANATION OF PLATES

FIG. 1.—Section of lymph node biopsied in Case 1. There is loss of normal architecture due to over-growth by lymphoma cells of the lymphocytic series. Reticulin Stain. × 390

FIG. 2.—(Chromosomes) Case 1. Karyotype from the peripheral blood showing 48 chromosomes with extra C group autosomes and a $Ph^1$ chromosome.

FIG. 3.—(Chromosome) Case 1. Karyotype showing 51 chromosomes with two $Ph^1$ + two extra C autosomes.

FIG. 4.—(Chromosomes) Case 1. Karyotype showing 49 chromosomes with two extra small metacentrics.

FIG. 5.—Peripheral blood. An atypical lymphocyte with deeply indented nucleus and scanty dark basophilic cytoplasm (centre). A normal lymphocyte is also shown. × 910

FIG. 6.—Lymph node biopsy from Case II. Prominent plasma cells are indicated with arrows. Tissue embedded in Maraglas and sectioned at 1 u. Toluidine blue stain. × 570

FIG. 7.—Portion of arterial wall from biopsy of subcutaneous nodule on forearm (Case II). There is a hyalinised thrombus occluding the lumen. There is fragmentation of the elastica (arrows).

FIG. 8.—Case 11. Tetraploid karyotype seen in the peripheral blood.

FIG. 9.—Case 11. Karyotype of one cell seen in the peripheral blood containing 47 chromosomes with an additional marker chromosome.

1

5

Adam, Thorburn, Gibbs, Brooks and Hanchard.

2

3

Adam, Thorburn, Gibbs, Brooks and Hanchard.

4

Adam, Thorburn, Gibbs, Brooks and Hanchard.

6

7

Adam, Thorburn, Gibbs, Brooks and Hanchard.

8

Adam, Thorburn, Gibbs, Brooks and Hanchard.

9

Adam, Thorburn, Gibbs, Brooks and Hanchard.

TABLE III.—*Nuclear Diameters*

A. *Normal lymphocytes*

| Nuclear diameter | | | | | | | | |
|---|---|---|---|---|---|---|---|---|
| (μ) . . . | 6·0 | 6·6 | 7·2 | 7·8 | 8·4 | 9·0 | 9·6 | 10·2 |
| No. of cells . . | 4 | 9 | 12 | 32 | 27 | 15 | — | 1 |

(a) Mean nuclear diameter 7·7 μ.

B. *Abnormal mononuclear cells*

| Nuclear diameter | | | | | | | | | | | | |
|---|---|---|---|---|---|---|---|---|---|---|---|---|
| (μ) . . . | 7·8 | 8·4 | 9·0 | 9·6 | 10·2 | 10·8 | 11·4 | 12·0 | 12·6 | 13·2 | 13·8 | >13·8* |
| No. of cells . . | 1 | 1 | 14 | 13 | 16 | 15 | 12 | 14 | 5 | 4 | 1 | 4 |

* Distributed as follows: 15 μ—3; 15·6 μ—1.

(b) Mean nuclear diameter 11·0 μ.

To date, this patient remains in clinical remission on prednisone, 20 mg. daily. In March, 1970, haematological and chromosomal studies indicated that the abnormal clone in the peripheral blood had diminished slightly (Table II), though two hyperdiploid cells (with 53 and 64 chromosomes) were found.

DISCUSSION

Lymphosarcoma has been clearly established on the basis of the clinical and histological features in Case I, though the precise date of onset is uncertain. It is debatable whether the exfoliative erythrodermia in January, 1968, was another example of the well-known association of this disorder with LRN (Montgomery, 1933), or was caused by penicillin, but it is interesting that abnormal mononuclear cells appeared in the peripheral blood several months before this or the other clinical features of lymphosarcoma became manifest. According to Dacie and Lewis (1968) these cells " indicate giant follicle lymphoma or lymphosarcoma " and we have noted similar cells in other patients with LRN. The question of a pre-existing AID is more doubtful. The decrease in serum globulin concentration (4·4 g.% in April, 1967, to 2·2 g.% in November, 1968) is noteworthy. Talal and Bunim (1964) demonstrated similar changes in their first case, who progressed from AID to LRN.

In Case II the most likely clinical diagnosis is an AID, probably polyarteritis nodosa. Abnormal mononuclear cells have been recognised in the peripheral blood from time to time, being prominent during relapse and absent during remission. The serum globulin (and presumably γ-globulin) concentration decreased from 7·8 g.% in February, 1968, to 4·1 g.% in January, 1969, as in our first case and that of Talal and Bunim. Hypogammaglobulinaemia is known to be associated with LRN (Ultman *et al.*, 1959; Miller, 1962). It seems reasonable to assume, therefore, that she has LRN though the clinical features are not yet obvious. The increase in the proportion of plasma cells in the bone marrow is secondary to the AID and/or the LRN: such reactive increase in plasma cells secondary to inflammatory and neoplastic disease is well-known, but is usually less than 10%. Myelomatosis was discounted because of the normal appearance of the plasma cells and the lack of other evidence clinically to support this diagnosis. This interpretation has been justified by her subsequent progress.

In both cases the evolution of an abnormal clone of cells was evident in the lymphocytic series in the peripheral blood. In Case I the abnormal clone seemed to have taken over completely in January, 1969, when it constituted 79% of the total white cell count and there were no lymphocytes in the peripheral blood. Could this have been an early phase in a " graft-versus-host " phenomenon? In Case II the abnormal clone represented almost two-thirds of the circulating lymphocytes but seemed to be present only as a small line in the marrow. (Chromosomal studies were not done on the marrow but the haematological findings suggest that the abnormal mononuclear cells were not a prominent constituent.) In both cases the abnormal cells were PHA responsive and did not appear to be dividing to any significant degree spontaneously. This situation is unusual in chronic myeloid leukaemia, chronic lymphocytic leukaemia and lymphomas (Spiers and Baikie, 1968*a* and *b*) where it is usually necessary to examine either the marrow or the lymph nodes to obtain sufficient dividing cells for chromosomal examination.

In Case I, clonal evolution followed the pattern described in the first model of de Grouchy *et al.* (1966) in which extra C chromosomes are acquired presumably by a series of non-disjunctional errors. The appearance of a chromosome resembling a $Ph^1$ is of great interest, as a diagnosis of chronic myeloid leukaemia (CML) was not considered at any time. de Nava *et al.* (1968) described two cases of CML and reviewed several others in which there was a reticulo-sarcoma-like process combined with the appearance of abnormal clones in peripheral blood cultures. These cases were also marked by the reduplication of the $Ph^1$ chromosome. They suggested that this phenomenon might accompany an increase in the invasive properties of the leukaemic cells resulting in infiltration of viscera, lymphadenopathy, and blastic crisis. In our case the origin of the $Ph^1$-like chromosome was not apparent but the duplication of this and other autosomes certainly accompanied the final phase of the patient's disease.

The cytogenetic abnormalities in Case II can be interpreted in two ways. Firstly, the tetraploid line might represent congenital mosaicism. We have not examined other tissues so we have no evidence for this. Only one previous case (Kohn *et al.*, 1967) of tetraploid-diploid mosaicism has been reported, in an infant with multiple congenital abnormalities, who only survived 8 months. The presence of a normal phenotype in our case is against this interpretation. The alternative, which we tend to favour, is that the tetraploidy represents a neoplastic or premalignant process. This is also a possibility in Kohn's case though there was no evidence of malignancy at autopsy. Kadowaki *et al.* (1965) reported a possibly similar situation in an infant with XX/XY chimaerism who developed terminally a " leukaemoid " stem line with karyotypic abnormalities. Against the neoplasia interpretation is that tetraploidy in lymphomas appears to be associated with an increased degree of malignancy and other karyotypic changes (Spiers and Baikie, 1968*b*).

Besides CML, clonal evolution has been reported in solid tumours (Baker, 1968; Goh, 1968*a*; Fraccaro *et al.*, 1968). In the canine venereal tumour, a remarkably consistent karyotype with a modal number around 59 has been found in four different parts of the world (Makino, 1963; Weber *et al.*, 1965; Barski and Cornefert-Jensen, 1966; Thorburn *et al.*, 1968). This tumour and the contagious reticulum cell sarcoma of the Syrian hamster, if they are in fact malignant tumours, may owe their consistent karyotype to the direct transfer of tumour cells between successive animals.

Both AID and LRN appear to occur more frequently in patients with constitutional chromosomal abnormalities such as mongolism and Klinefelter's syndrome (Fialkow, 1966; Miller, 1966) and have been described in patients with balanced and unbalanced translocation (Buchanan *et al.*, 1967; Goh, 1968*b*; Zuelzer *et al.*, 1968).

The occurrence of such cases as ours is unusual. If it is accepted that AID was present in Case I as well as LRN and, in Case II, a neoplasm as well as AID, then they might provide a further link in the auto-immunity-neoplasia complex. As Dameshek (1966) said "one may speculate that in both AID and leukaemia an abnormal clone of immunocytes has developed whether by mutation or other mechanism " (? deletion of part of a chromosome). " The clone becomes enlarged because self antigens coming to it are not recognised by the abnormal immunocytes which proliferate. This may result in an immunoproliferative response (auto-immunity), in a localised (lympho-sarcomatous) process or a generalised (leukaemic) disease." This was in keeping with the hypothesis of Kaplan and Smithers (1959) who suggested that autologous tumour cells have undergone antigen deletion which means that they would attack normal lymphocytes bearing such antigens. Case I fits these theories much better than Case II. The former showed a $Ph^1$-like chromosome as well as additional autosomes, though the tumour cells remained antigenically responsive. Case II showing tetraploidy, was clinically a much less malignant condition though further karyotypic evolution may occur at a later date in transformation to more obvious neoplastic disease.

We would like to thank our many colleagues for their assistance, Miss P. A. Martin for some of the chromosome analyses, Mr. and Mrs. E. Dawson for the photography and Professor G. Bras for helpful criticism. The cytogenetic work was supported by grants from the Wellcome Trust and the Standing Advisory Committee for Medical Research in the British Caribbean. Boots Pure Drug Company supplied the heparin.

### REFERENCES

ARAKAKI, D. T. AND SPARKES, R. S.—(1963) *Cytogenetics*, **2**, 57.

BAKER, M. C.—(1968) *Br. J. Cancer*, **22**, 683.

BARSKI, G. AND CORNEFERT-JENSEN, FR.—(1966). *J. natn. Cancer Inst.*, **37**, 787.

BUCHANAN, J. G., SCOTT, P. J., MCLACHLAN, E. M., SMITH, F., RICHMOND, D. E. AND NORTH, J. D. K.—(1967) *Am. J. Med.*, **42**, 1003.

CAMMARATA, R. J., RODNAM, G. P. AND JENSEN, W. N.—(1963) *Archs intern. Med.*, **111**, 330.

DACIE, J. V. AND LEWIS, S. M.—(1968) ' Practical Haematology ', 4th edition. London (Churchill), p. 155.

DAMESHEK, W.—(1966) *Lancet*, i, 1968.

DE GROUCHY, J., DE NAVA, C., CANTU, J. M., BILSKI-PASQUIER, G. AND BOUSSER, J.—(1966) *Am. J. hum. Genet.*, **18**, 485.

DE NAVA, C., DE GROUCHY, J., THOYER, C., BOUSSER, J., BILSKI-PASQUIER, J. AND FRETEAUX, J.—(1968) *Annls Génét.*, **12**, 83.

FIALKOW, P. J.—(1966) *Am. J. hum. Genet.*, **18**, 93.

FRACCARO, M., MANNINI, A., TIEPOLO, L., GERLI, M. AND ZARA, C.—(1968) *Lancet*, i, 613.

GOH, K-O.—(1968*a*) *Archs intern. Med.*, **122**, 241.—(1968*b*) *Am. J. Dis. Child.*, **115**, 732.

HARGREAVES, M. M.—(1962) *J. Ark. med. Soc.*, **58**, 522.

ISRASENA, T., QUATRALE, A. C. AND BECKER, K. L.—(1967) *Lancet*, ii, 1226.

KADOWAKI, J., THOMPSON, R. I., ZUELZER, W. W., WOOLLEY, P. V., BROUGH, A. J. AND GRUBER, D.—(1965) *Lancet*, ii, 1152.

KAPLAN, H. S. AND SMITHERS, D. W.—(1959) *Lancet* ii, 1.

KOHN, G., MAYALL, B. H., MILLER, M. E. AND MELLMAN, W. J.—(1967) *Pediat. Res.*, **1**, 461.
LEA, A. J.—(1964) *Ann. rheum. Dis.*, **23**, 480.
MAKINO, S.—(1963) *Ann. N.Y. Acad. Sci.*, **54**, 1197.
MILLER, D. G.—(1962) *Ann. intern. Med.*, **57**, 703.
MILLER, R. W.—(1966) *New Engl. J. Med.*, **275**, 87.
MONTGOMERY, H.—(1933) *Archs Derm. Syph.*, **27**, 253.
RAZIS, D. V., DIAMOND, H. D. AND CRAVER, L. F.—(1959) *Am. J. med. Sci.*, **238**, 327.
SCHWARTZ, R. S. AND BELDOTTI, L.—(1965) *Science, N.Y.*, **149**, 1511.
SPIERS, A. S. D. AND BAIKIE, A. G.—(1968*a*) *Br. J. Cancer*, **22**, 192.—(1968*b*) *Cancer, N.Y.*, **22**, 193.
STANLEY, N. F.—(1966) *Lancet*, i, 961.
TALAL, N. AND BUNIM, J. J.—(1964) *Am. J. Med.*, **36**, 529.
THORBURN, M. J., GWYNN, R. V. R., RAGBEER, M. S. AND LEE, B. I.—(1968) *Br. J. Cancer*, **22**, 720.
ULTMAN, J. E., FISH, W., OSSERMAN, E. AND GELHORN, A.—(1959) *Ann. intern. Med.*, **51**, 501.
WEBER, W. T., NOWELL, P. C. AND HARE, W. C. D.—(1965) *J. natn. Cancer Inst.*, **35**, 537.
ZUELZER, W. W., THOMPSON, R. I. AND MASTRANGELO, R.—(1968) *J. Pediat.*, **72**, 367.